# Exposure to Cerium Dioxide Nanoparticles Differently Affect Swimming Performance and Survival in Two Daphnid Species

**DOI:** 10.1371/journal.pone.0071260

**Published:** 2013-08-15

**Authors:** Ester Artells, Julien Issartel, Mélanie Auffan, Daniel Borschneck, Antoine Thill, Marie Tella, Lenka Brousset, Jérôme Rose, Jean-Yves Bottero, Alain Thiéry

**Affiliations:** 1 Institut Méditerranéen de Biodiversité et d’Ecologie marine et continentale, IMBE UMR-CNRS 7263, Aix-Marseille Université, Marseille, France; 2 Institut Méditerranéen de Biodiversité et d’Ecologie marine et continentale, IMBE UMR-CNRS 7263, Aix-Marseille Université, Aix-en-Provence, France; 3 Centre Européen de Recherche et d’Enseignement des Géosciences de l’Environnement, CEREGE UMR-CNRS 7330, Aix-Marseille Université, Aix-en-Provence, France; 4 iCEINT, International Consortium for the Environmental Implications of Nanotechnology, Aix-en-Provence, France; 5 Comissariat à l’énergie atomique et aux énergies alternatives, CEA Saclay, IRAMIS, UMR 3299, Laboratoire Interdisciplinaire sur l’Organisation Nanométrique et Supramoléculaire, Gif-sur-Yvette, France; 6 Labex SERENADE 2012 “*Safer Ecodesign Research and Education applied to NAnomaterial Development*”, Aix-en-Provence, France; Dowling College, United States of America

## Abstract

The CeO_2_ NPs are increasingly used in industry but the environmental release of these NPs and their subsequent behavior and biological effects are currently unclear. This study evaluates for the first time the effects of CeO_2_ NPs on the survival and the swimming performance of two cladoceran species, *Daphnia similis* and *Daphnia pulex* after 1, 10 and 100 mg.L^−1^ CeO_2_ exposures for 48 h. Acute toxicity bioassays were performed to determine EC_50_ of exposed daphnids. Video-recorded swimming behavior of both daphnids was used to measure swimming speeds after various exposures to aggregated CeO_2_ NPs. The acute ecotoxicity showed that *D. similis* is 350 times more sensitive to CeO_2_ NPs than *D. pulex,* showing 48-h EC_50_ of 0.26 mg.L^−1^ and 91.79 mg.L^−1^, respectively. Both species interacted with CeO_2_ NPs (adsorption), but much more strongly in the case of *D. similis*. Swimming velocities (SV) were differently and significantly affected by CeO_2_ NPs for both species. A 48-h exposure to 1 mg.L^−1^ induced a decrease of 30% and 40% of the SV in *D. pulex* and *D. similis*, respectively. However at higher concentrations, the SV of *D. similis* was more impacted (60% off for 10 mg.L^−1^ and 100 mg.L^−1^) than the one of *D. pulex*. These interspecific toxic effects of CeO_2_ NPs are explained by morphological variations such as the presence of reliefs on the cuticle and a longer distal spine in *D. similis* acting as traps for the CeO_2_ aggregates. In addition, *D. similis* has a mean SV double that of *D. pulex* and thus initially collides with twice more NPs aggregates. The ecotoxicological consequences on the behavior and physiology of a CeO_2_ NPs exposure in daphnids are discussed.

## Introduction

To date, the effects of CeO_2_ nanoparticles (NPs) on aquatic and terrestrial environments are of growing concern since their production and uses are expected to rise in the future [1]. The CeO_2_ NPs are increasingly used in industry (as oxidation catalyst, gas sensor, polishing materials, UV absorber). These applications rely on the remarkable properties of Ce such as, its high affinity to oxygen, a potential redox chemistry involving Ce(III)/Ce(IV) and its unique adsorption/excitation energy bands [2]. However, the environmental release of these NPs, and subsequent behavior and biological effects are currently unclear. Consequently, since 2008 [3] CeO_2_ NPs have been included the OECD list of nanomaterials requesting immediate testing.

Understanding the toxic effects of these emerging xenobiotics is therefore crucial in order to anticipate the consequences of the potential degradation of ecosystems [4], [5] and their potential impact on health. The biotopes of aquatic organisms constitute the major sink for pollutants that accumulate the inputs from the surrounding hydrographic basins. Consequently, aquatic organisms, especially in the vicinity of urbanized areas, are generally considered as highly vulnerable. Studying the potential toxic effect of emerging xenobiotics of NPs on these vulnerable environments is a more than reasonable strategy.

Over the past few years, many studies have attempted to decipher the cellular toxic effects of NPs in aquatic organisms. It is now widely recognized that one of the major harmful aspects of these substances lies in the oxidative stress they induce [6]. Indeed, exposure of aquatic organisms to metallic NPs such as Fe-NPs [7] TiO_2_, CuO/Cu_2_O and Ag-NPs [8]–[11] as well as carbon nanomaterial such as fullerene [12], [13]; and also silica NPs [14] has been correlated to an increase in oxidative damages and to a modification of the antioxidant system [8]–[13]. In addition to oxidative stress markers, a large battery of other ecotoxicological endpoints has been monitored in aquatic organisms exposed to NPs. Among them, it was shown that some NPs induce the expression of varous defense cellular biomarkers such as heat shock proteins (e.g. in *D. magna* exposed to Cu_2_O NPs [9]), metallothioneins (e.g. in *S. plana* after a CuO NPs exposure [10]), or detoxification complexes such as CYP family isozymes (e.g. in Ag-NPs exposed medaka and C60-exposed fathead minnow [15], [16]). At a larger scale, some NPs can also induce histological abnormalities as observed in the medaka gills after exposure to Fe-NPs [7]. These fundamental sub-individual toxic effects are thought to be responsible for the time/concentration dependant-mortality observed after NPs exposure of aquatic animals. Although these case-by-case studies in highly controlled conditions are important to identify and understand the ecotoxicity mechanisms at the sub-individual scale (i.e. cellular and molecular levels), it is necessary to go further and to study the ecotoxicity of NPs at a larger biological scale. This will allow translating the toxic effects observed on sub-individual or individuals into relevant information to predict consequences at population levels. In this regards, modifications of behavior [5], [17] could be a good indicator. Indeed, behavioral parameters are accurate and reliable indicators since the behavior of an organism is the endpoint of a sequence of complex neurophysiological events (stimulation of neurons via the release of chemical messages, muscular contractions) [18]–[20]. Behavioral response could therefore be a very sensitive indicators of stress and very useful in obtaining a realistic picture of the effects of contaminants at the ecosystem level.

In aquatic organisms, swimming behavior responses to several environmental stimuli have been intensively investigated [21]–[23], especially in the case of permanently swimming zooplankters like daphnids. The swimming of these organisms is closely related to the energetic metabolism and to ecological parameters as food intake, predator escape and reproduction [24]. While the swimming of daphnids has frequently been used to test different substances such as, constituents of oral pill [25] **,** natural cyanobacteria toxins produced by algal blooms [21], [26], [27], metals contaminants as cadmium [28], [29], copper [20], and organic xenobiotics as PCB, tributyltinchloride [30], cypermethrin [31], only few studies deal with nanoparticle effects. To our knowledge, only fullerene (nC_60_), TiO_2_ and Ag NPs were tested in relation to the swimming behavior of daphnids [32]–[36].

The present study is part of a series of tasks required to understand the impact of new nanotechnologies on the environment [37] **.** We propose to evaluate the CeO_2_ NPs impact on both the survival and swimming behavior of two daphnid species. To date, most of the ecotoxicity studies of NPs were performed with a single-species approach whereas a comparative multi-species approach provides a more complete and ecologically relevant overview of the impact of NPs in the ecosystem [9], [17], [32]–[36], [38], [39]. The Anomopod (Cladocera) *Daphnia pulex* (L., 1758) is an ecologically and genetically well-known organism [40], [41] and a good model to study multi-stressors in freshwater environments. For compaison with a closely related other species, the experiment was also conducted in *Daphnia* (*Ctenodaphnia) similis* (Claus, 1876), a water flea species present in temporary lakes in Provence (France).

Using an original experimental approach, our study revealed that both daphnids were differentially impacted by NPs exposure, bringing new information on the toxic effects of CeO_2_ NPs.

## Materials and Methods

### 2.1. Nanoparticles Characterization

The CeO_2_ NPs were provided as a stable suspension at 130 g.L^−1^ of CeO_2_ by Rhodia Chemicals**®**. The size and crystalline structure of CeO_2_ NPs were determined using a Transmission Electron Microscope (TEM) JEOL® JEM 2010F URP22 equipped with an X-ray EDS-Kevex detector and an ELS-Gatan imaging filter. Samples (n = 60) were prepared by evaporating a droplet of a CeO_2_ NPs suspension on a carbon-coated copper grid at ambient temperature. The aggregation state of CeO_2_ NPs was characterized in the natural water (Cristaline**®**) used for daphnia cultures using the granulometer Malvern3000 (Malvern Instruments®, UK).

### 2.2. Organisms Breeding

*Daphnia pulex* (*D. pulex*) were collected from a permanent pond in the Paris countryside, the *Forêt de Sordun*, in the Seine and Marne Region, (48° 31′ 51″N, 3° 24′ 61″E, 175 m a.s.l.) and *Daphnia (Ctenodaphnia) similis* (*D. similis*) were collected, in January 2012, from a temporary pond, the *Mare de Saint Maximin,* in the Var Region, in Southern France (43° 26′ 16″N, 5° 52′ 19″E, 298 a.s.l.) in January 2012. No specific permissions were required for these locations. We confirm that the field studies did not involve endangered or protected species. Both species were acclimated and bred in the laboratory at 20±2°C with a natural photoperiod (10 h Light, 14 h Dark), and fed daily with the freshwater unicellular *Chlorella vulgaris* (Beijerinck, 1890) (AC149 strain, Algobank, France) at a concentration of 10^5^–10^6^ cells.mL^−1^. The breeding procedure was adapted from Barata [42]. The nutritive solution was the commercialized natural water (Cristaline**®**, France) (pH 8.5, 290 mg.L^−1^ HCO_3_^−^, 5 mg.L^−1^ SO_4_^2−^, 4 mg.L^−1^ Cl^−^, 39 mg.L^−1^ Ca^2+^, 25 mg.L^−1^ Mg^2+^, 19 mg.L^−1^ Na^+^, 1.5 mg.L^−1^ K^+^).

### 2.3. Acute Toxicity Assay

The acute toxicity tests were conducted in accordance with OECD guideline number 202 [43] **,** compatible with the procedure proposed by the US-EPA [44]. The concentrations used in this study are based on the EC_50_ from CeO_2_ exposed *Daphnia magna* [45]. The test medium was prepared from a 130 g.L^−1^ CeO_2_ NPs original stock solution diluted in miliQ water to obtain a final CeO_2_ NPs solution. To 2.5 ml of this final solution was then added to 47.5 ml of rearing Cristaline® water to obtain the experimental concentration used for the test. The bioassays were performed in septuplicate with five 8 days-old organisms. Eight days-old daphnids were chosen in order to minimize confounding effects of growth and reproduction energetic cost of younger and older stages, respectively [46]. Daphnids were placed into 50 mL of test medium and exposed for 96 h to 0, 0.1, 1, 10, 50 and 100 mg.L^−1^ CeO_2_ NPs. Immobility and mortality data were recorded each 24 h. The CeO_2_ NPs concentration in each chamber during toxicity test is considered constant as evaporation was negligible.

### 2.4. Swimming Velocity Assay

The effects of CeO_2_ NPs on *D. pulex* and *D. similis* swimming velocity were investigated. Both species were exposed to 0, 1, 10 and 100 mg.L^−1^ CeO_2_ NPs for 48 h in glass vials (45 mm diameter) containing 50 mL of solution. We used 3 replicates for each exposure conditions: each replicate consisted in at least 4 surviving daphnids in a vial. As both species were unable to move vertically at concentrations higher than 1 mg.L^−1^, only horizontal movements were measured. Before recording the daphnid movements, the volume of culture medium was slowly and carefully adjusted to 10 ml in order to limit vertical movement of daphnids. Daphnid movements were recorded using a Cam Sport® camera (China) EVO model operating at 25 frames.s^−1^ and high resolution 736×480 pixels; the camera was placed 15 cm above the swimming chamber. For each replicate and exposure concentration, 1 minute sequences were recorded and then transferred to a computer for analysis. Individual swimming velocities were calculated on the basis of a 10 seconds travel using ImageJ 1.46 and MTrackJ plugin, which allows calculating the distance traveled by the daphnid between two frames (*i.e.* 41.7 ms).

### 2.5. Micro-X-ray Fluorescence Analysis

The Ce spatial distribution in daphnids was determined with the XGT^7000^ X-ray analytical miscroscope (Horiba® Jobin Yvon) equipped with an X-ray tube producing a high-intensity beam with a 10 µm spot size (Rh X-ray source, 30 kV, 1 mA, equipped with an EDS detector). *D. pulex* and *D. similis* exposed to 10 mg.L^−1^ of CeO_2_ NPs for 48 h were analyzed using a Peltier freezing system to maintain the sample frozen during analysis. Given that the X-ray beam completely penetrates the sample, the obtained chemical images are 2D projections of a 3D sample. Elements from Na to U can be detected with a sensitivity range from about 50 mg.kg^−1^ to a few percent mass depending on the atomic number of the element and the nature of the matrix.

### 2.6. Statistical Analysis

The data obtained in these acute toxicity tests were used in order to determine the Median Effective Dose (EC_50_); this is done through Probit analyses using the statistical package SPSS (version 20, IBM®). For the swimming velocity statistical analysis, the normality of the data and the homogeneity of variances were verified using the Kolmogorov-Smirnov test and the Levene’s test, respectively. Differences between the mean swimming velocities of the control and the exposed groups were assessed using a one-way ANOVA. When significant differences were found, a Tukey post-hoc test was performed. Statistical analyses were performed using Statistica 6 (StatSoft Inc., Tulsa, USA). A 5% (p<0.05) significance was used in all tests.

## Results

### 3.1. Nanoparticles Physico-chemical Behavior

**Figure 1:**
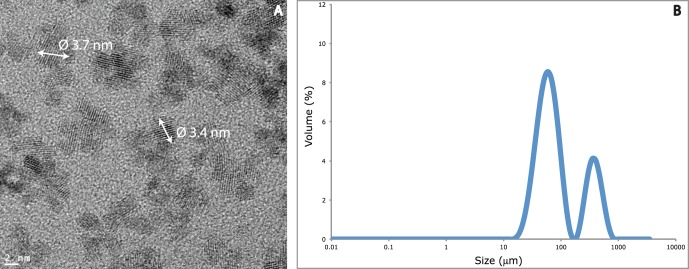
Physico-chemical characterization of the CeO_2_ NPs. TEM picture of the CeO_2_ in deionized water (A) and distribution of the hydrodynamic diameters within daphnia medium (B).

By TEM, we observed well-crystallized clusters of cerianite (95–98% of purity) with a d-spacing (∼3.2 Å) close to the d_111_ of CeO_2_ (d_hkl_). These clusters are pseudo-spherical with a diameter of 3±1 nm (n = 60) (Fig. 1). In pure water, these CeO_2_ NPs (100 mg.L^−1^) are colloidally stable with a negative zeta potential (−40±5 mV at pH 4) and an average hydrodynamic diameters of ∼ 8 nm. Based on this value, the specific surface area of the CeO_2_ NPs was calculated to be about 110 m^2^.g^−1^.

The natural water (Cristalline®) used in the exposure scenario is at pH = 8.5 and elevated ionic strength. Once injected in the natural water, NPs aggregated due to the neutralization of the surface charges by the salts and the pH which is close to the isoelectric point (PIE) of our material. The PIE of these CeO_2_ NPs in water has previously been measured to be 7.5–8 [47]; their zeta potential measured in natural water is low, −10±2 mV (pH 8.5). Figure 1B shows the aggregate size distribution of a 100 mg.L^−1^ CeO_2_ NPs suspension in natural water measured 25 min. after NPs injection. Such a distribution of hydrodynamic diameters is not representative of the real size distribution of the NPs aggregates as the data treatment does not take into account the specific scattering properties of the NPs fractal aggregates. However, it clearly shows that CeO_2_ NPs form large aggregates with a maximum size larger than 300 µm.

### 3.2. Ecotoxicity Testing of CeO_2_ NPs Towards *D. pulex* and *D. similis*

**Figure 2:**
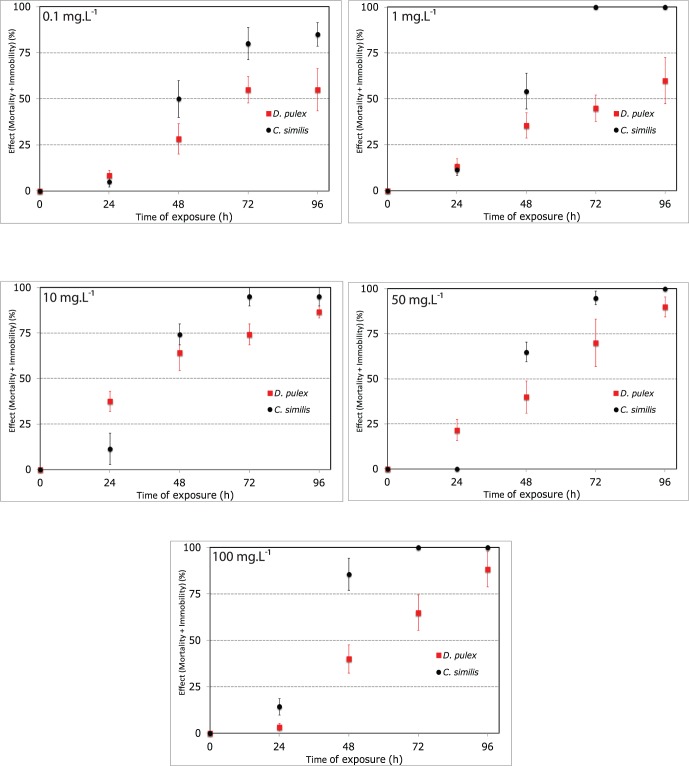
Effect curve *vs* time of *D. similis* and *D. pulex* at 0.1 mg.L^−1^, 1 mg.L^−1^, 10 mg.L^−1^, 50 mg.L^−1^ and 100 mg.L^−1^ of CeO_2_ NPs. Values are Mean EC_50_±SD.

The acute ecotoxicity study showed that *D. similis* was more sensitive to CeO_2_ NPs than *D. pulex*. For both *D. pulex and D. similis,* the toxic effects increased with increasing exposure duration. During the first 24 h, *D. pulex* was significantly more affected by CeO_2_ NPs than *D. similis*, but after 48 h an opposite trend occurred with *D. similis* displaying higher immobility and mortality values (Fig. 2). In the 100 mg.L^−1^ treatment, *D. similis* was more affected by CeO_2_ NPs than *D. pulex* during all test periode. The 48-h EC_50_ for *D. similis* were calculated to be 0.26 mg.L^−1^. For *D. pulex*, the 48-h EC_50_ (91.79 mg.L^−1^) obtained was 350 times higher than the 48-h EC_50_ of *D. similis.* After 72 h, surviving specimens were only observed for *D. pulex* in all of concentrations treatment while for *D. similis* in 0.1 mg.L^−1^ only few surviving specimens are found. This data is not sufficient to calculate the 72-h and 96-h EC_50_ of *D. similis*. The 72-h EC_50_ and 96-h EC_50_ for *D. pulex* were respectively 0.94 mg.L^−1^ and 0.78 mg.L^−1^.

### 3.3. Relation Nanoparticules/Cuticle

**Figure 3:**
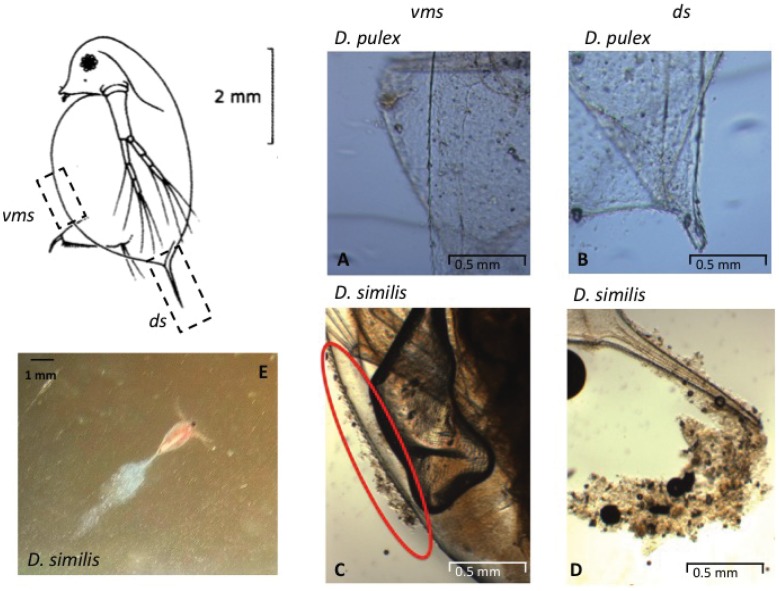
Representative image of distal spine (ds) and ventral margin of the shield (vms) in *D. pulex* and *Daphnia similis* exposed to 10 mg^.^L^−1^ of CeO_2_ NPs for 48 h. Note the accumulation of particles onto the cuticle of *D. similis*. The optical image (E) represents the *D. similis* after 48 h exposure to 10 mg.L^−1^ of CeO_2_ NPs.

*D. similis* and *D. pulex* present distinct morphologies. *D. similis* have a large distal spine (0.6–1 mm) and many small spines on the cuticle (Fig. 3C and D). On the opposite, *D. pulex* displays a short distal spine (0.10–0.25 mm) and only few spines on the cuticle (Fig. 3A and B). Using optical microscopy, we noticed that depending on their morphology, these daphnids were able to accumulate particles onto their shield following CeO_2_ NPs treatment. After a 48 h of exposure to 10 mg.L^−1^ of CeO_2_ NPs, *D. similis* accumulated a significant amount of particles onto the distal spine (Fig. 3D) and onto specific areas of the carapace (Fig. 3C), whereas no or only very slight accumulation was observed with *D. pulex* (Fig. 3A and B). This accumulation of particles formed a cloud just behind the distal spine when *D. similis* swam (Fig. 3E).

**Figure 4:**
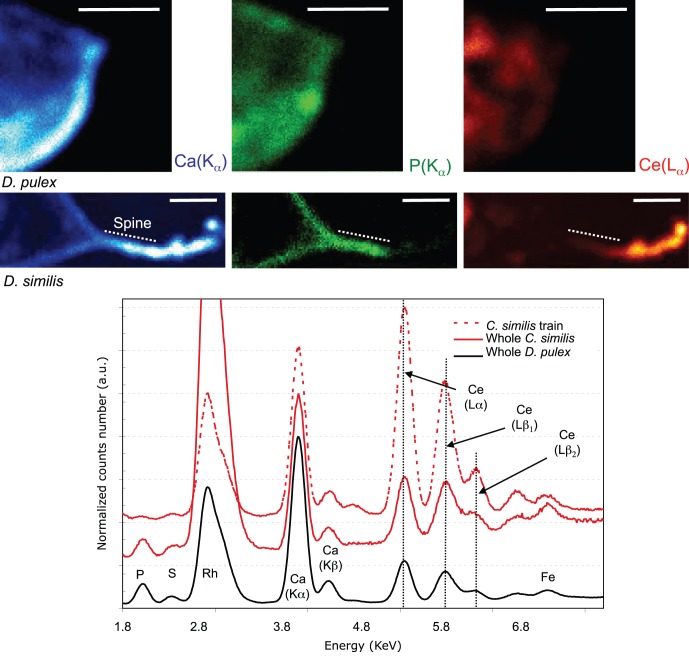
Distribution of Ce (Lα line), P (Kα line) and Ca (Kα line) on the posterior region of *D. pulex* and *D. similis* exposed 48 h to CeO_2_ NPs. Chemical map parameters: 128 pixel^2^ image, 1 pixel: 8 µm, total counting time 20000: sec, scale (white bar): 500 µm. Mean XRF spectra corresponding to specific area of the individual were generated from the hyperspectral map.

Micro-XRF was used to identify the chemical composition of this cloud. Due to the presence of calcium and phosphorous, it is possible to observe the cuticle and the distal spine of daphnids on the Ca and P map (Fig. 4, Ca and P maps). Using the P map, we measured the length of the distal spine of *D. similis* to be 600 µm. This value was similar to the length measured by optical microscopy. After an incubation of 48 h, Ce was detected in a line just behind the distal spine in *D. similis* and on the surface in both species (Fig. 4). This CeO_2_ line is only visible in the case of *D. similis* and corresponds to the cloud observed using optical microscopy (Fig. 3).

### 3.4. Swimming Velocity

**Figure 5:**
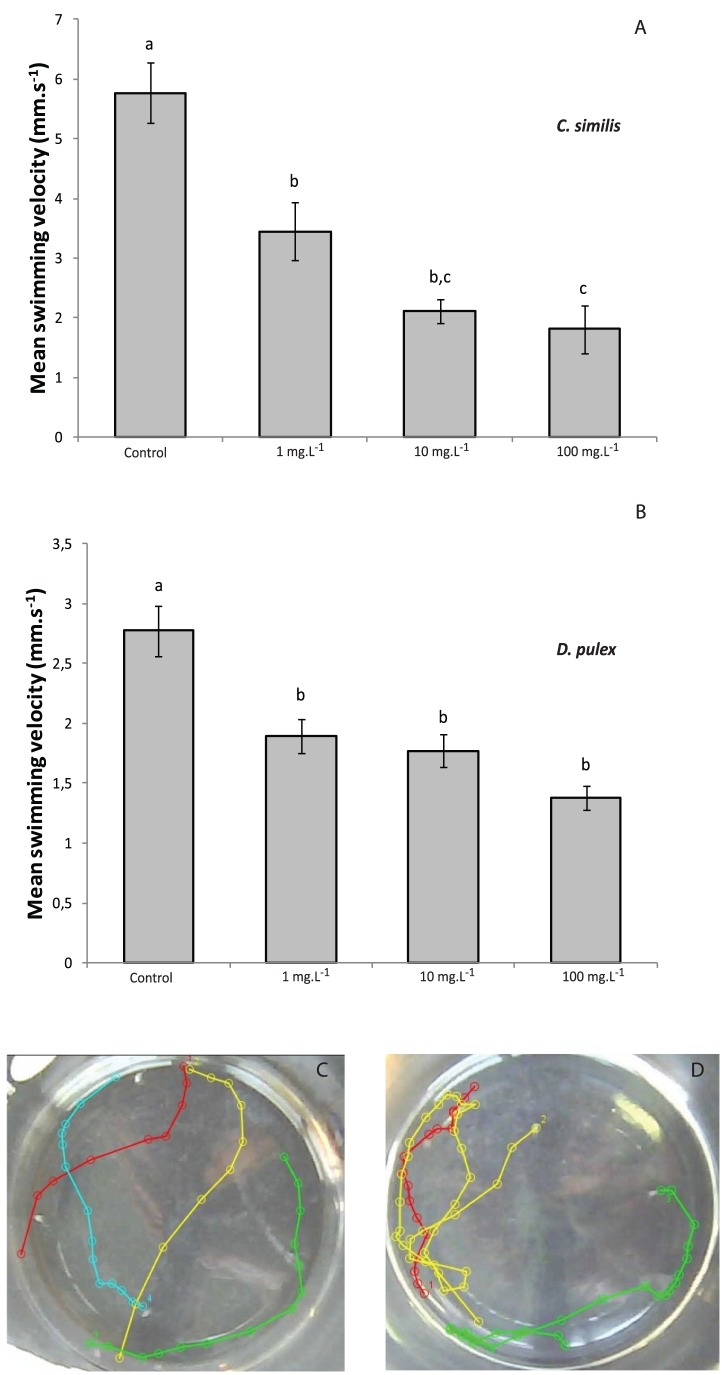
Mean swimming velocity in *D. similis* (A) and *D. pulex* (B) exposed to CeO_2_ NPs for 48 h. Values are means ± SEM. Letters show significant differences established by one-way ANOVA and Tukey *post-hoc* (*p*<0.05). *D. similis* swimming tracks in control (C) and after a 48-h exposure to 10 mg.L^−1^ of CeO_2_ NPs (D).

Due to the strong interactions between CeO_2_ NPs and the cuticle, we examined the ability of daphnids to swim in these contaminated exposure media. Figure 5 shows that the average swimming velocities (SV) were differently and significantly affected by CeO_2_ NPs for both species *i.e.* exposed daphnids swam slower than non-exposed daphnids of similar size. After 48-h exposure to 1 mg.L^−1^, a decrease of 30% and 40% of the SV is measured for *D. pulex* and *D. similis*, respectively. However at higher concentrations, the SV of *D. similis* was more impacted (60% off for 10 mg.L^−1^ and 100 mg.L^−1^) than the one of *D. pulex*. While the SV was significantly altered, no change of the hop frequency -i.e. number of downward thrusting of the second antennae below the helmet and then back above per minute- was observed in both species after a 48-h exposure to CeO_2_ NPs.

## Discussion

### 4.1 NPs Aggregation Kinetics versus NPs/Daphnids Interaction Kinetics

The ∼8 nm CeO_2_ NPs (hydrodynamic diameter) are introduced in a natural water at a pH close to their PIE and a ionic strength of 1.4 10^−2^ mol.L^−1^. In such physico-chemical conditions the repulsive electrostatic interactions which contribute to the colloidal stability of the CeO_2_ NPs are sufficiently reduced to trigger fast aggregation. Assuming a purely Brownian mechanism for the NPs collisions, it is possible to estimate the half life (t_1/2_) of fully destabilized NPs at a concentration of 100 mg.L^−1^ which depends on the temperature (T), viscosity (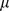) and initial NPs number concentration (C_0_) as:
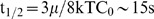



This simple calculation shows that even if a significant residual stabilization is active, the NPs will aggregate very quickly. The size distribution represented on figure 1B after 25 min. is most probably reached at the very beginning of the experiment.

When the NPs interact with daphnids, the relevant collision mechanism is no longer the Brownian motion of the NPs. The active motion of the daphnids increases their collision rate with the NPs. A simple estimate of the ratio between the collision due to the Brownian motion of the NPs and those due the active swimming motion of the daphnids can be evaluated. First, Brownian collisions frequencies (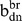) involved between both the NPs and the daphnids can be written as 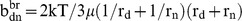, where 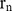 is the radius of a NP and 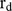 is the radius of a daphnid. As 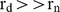, the equation can be simplified to 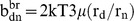.

As to the collisions induced by the active motion of the daphnids, it is possible to assume that the motion of a daphnid is equivalent to a shear gradient (G) given by 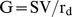. Assuming this shear gradient, the collision frequency between the NPs and the daphnids reads as:
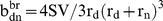



Using again the fact that r_d_>>r_n_, we have 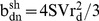.

Using these simplified expressions, we have 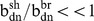 in the whole range of possible swimming velocities. The only important collision mechanism is thus the collisions induced by the swimming motion of the daphnids in the aggregated NPs suspension. As the size of the daphnids is the same for the two species, the difference in collision frequencies only depends on the differences of swimming velocities. Thus, we can conclude that initially *D. similis* collide with twice more aggregates than *D. pulex*.

### 4.2. Relation between Daphnia Morphology and the uptake of CeO_2_ NPs

Low levels of NPs adsorption to the exoskeleton of aquatic invertebrates has already been observed in a few previous studies (see e.g. *D. magna* exposed to nC_60_, TiO_2_ and Ag NPs [35], [36], [48] and *Ceriodaphnia dubia* exposed to Quantum Dots [49]). In a recent study, Gaiser *et al.* [50] observed a very slight adsorption of CeO_2_ NPs on *D. magna* neonates’ cuticles after 96 h of exposure to 10 mg.L^−1^. These different clinging capacities of CeO_2_ NPs may be due to their physico-chemical characteristics such as size, chemical nature, or surface coating [50]. The mechanisms of interaction between NPs and the cuticle are however not clear. In our case, *D. pulex* and *D. similis* display different accumulation of CeO_2_ NPs onto their cuticle. *D. similis* accumulates large aggregates whereas *D. pulex* is only slightly covered by small NPs or NPs aggregates. The objective of this section of the discussion is to understand the possible origin of these differences.

The interaction between the CeO_2_ NPs and the cuticle observed can be discussed in terms of both physico-chemical and mechanical processes. Indeed to accumulate on the cuticles of daphnids, NPs have first to undergo a collision with the cuticle; the frequency at which this occurs depends on various mechanical processes, as for example viscosity of the fluid, relative size of the aggregates and the daphnids and swimming velocities of the daphnids. Then, once on the surface of the daphnids, the NPs or the NPs aggregates can only accumulate if they adhere sufficiently strongly to resist the viscous strain induced by the daphnids active swimming motion.

A micro crustacean cuticle is mostly composed of a fibrous phase of crystalline chitin (nanofibrils with 3 nm of diameter), sugars, silk-like proteins attached through specific H-bonds, and globular proteins, which confer a net negative surface charge at neutral pH [51]. In our experimental conditions, a zeta potential of −10±2 mV was measured at the surface of the CeO_2_ NPs (at pH 8.5). This zeta potential value corresponds to a global negative charge which should generate a long distance repulsive potential between the NPs aggregates and the cuticle. At shorter distances, van der Waals attraction and possible surface complexation at specific CeO_2_ sites can be responsible for the NPs adhesion. Indeed, the surface of the CeO_2_ NPs being composed by a mixture of positive and negative sites, it is likely that mechanisms associating steric effects and surface complexation (with thiolated or carboxilated groups…) between the cuticle and the surface of CeO_2_ NPs contribute to the short distance adhesion. While, these physico-chemical interactions between CeO_2_ NPs and the cuticle (governed by van der Waals, steric effects and surface interaction) should be similar for both species, differences in morphology between *D. similis* and *D. pulex* are possibly responsible for different mechanical trapping of NPs or NPs aggregates. The ability to regain normal mobility after molting [39] has not been considered here as during our experiments the daphnids did not molt.

The main differences between the two daphnids species are the initial swimming velocity and the morphology of the cuticle surface. Due to its higher initial swimming velocity, the *D. similis* collide with NPs at an initial rate twice more important than the one of *D. pulex*. Moreover, the surface of *D. similis* is covered with several spines and has a long distal spine, while *D. pulex* has a short distal spine and very few spines on the cuticle. All the spines around the cuticle of *D. similis* and especially the distal spine generate reliefs that can act as traps for the CeO_2_ large NPs aggregates which dominate in the exposure media. These morphological differences may also modify the resistance of the trapped NPs aggregates against viscous strain due to the fluid motion. Furthermore, due to its smoothest surface, *D. pulex* will only retain the smaller aggregates.

Consequently, while *D. similis* is able to mechanically trap the dominating population of large NPs aggregates, *D. pulex* is only able to physico-chemically adsorb small aggregates. The proportion of these small agregates is not known quantitatively, but most probably it only represents a minor part of the aggregates population.

### 4.3. An Interspecific Sensitivity to NPs

In this study, the two different daphnids species present drastically different EC_50_. Interestingly, *D. similis* has a lower 24 h EC_50_ and a larger 48-h or more EC_50_ compared to *D. pulex*. *D. similis* also displays a large CeO_2_ adsorption/accumulation on its cuticle under the form of large aggregates and a high decrease of its SV. In contrast, *D. pulex* presents a high 24 h EC_5O_, a small CeO_2_ adsorption/accumulation under the form of smaller aggregates and a low decrease of the SV. The comparison with the EC_50_ values available for TiO_2_ NPs in the literature reveals strong interspecific survival differences in exposed daphnids (see Table 1). However, these different toxicities might be due to either different physico-chemical properties of TiO_2_ NPs or exposure conditions. In the current work, the same CeO_2_ NPs and exposure conditions were used for both species. Consequently, the different toxic effects of CeO_2_ NPs between *D. similis* and *D. pulex* reflect different sensibilities of each species. In daphnids, the toxicity of CeO_2_ NPs can be exerted via two ways: a mechanical toxicity by adsorption/accumulation of large NPs aggregates on the cuticle, and/or a metabolic toxicity by internalization of CeO_2_ NPs into the cells. In aquatic organisms, potential routes of internalization include entry across gills, olfactory organs or gut epithelium [14]. Although Auffan *et al.* (2013) showed that CeO_2_ NPs accumulate in the digestive tract of *D. pulex* [39], the metabolic toxicity of CeO_2_ NPs in daphnids is still unclear and, as far as we know, no direct evidence of internalization has been found in these organisms. However, in vitro studies on vertebrate cell cultures showed that the CeO_2_ NPs can penetrate into cells and induce oxidative stress [52], [53]. Further studies are needed to decipher the metabolic toxicity of CeO_2_ NPs in aquatic invertebrates.

**Table 1:** Median, maximal and minimal values of 48-h L(E)C_50_ of daphnids species tested with TiO_2_ NPs calculated from differents studies [38], [58]–[72].

Species	Median L(E)C_50_	Max L(E)C_50_	Min L(E)C_50_
*Daphnia magna*	23.55	20000.00	NA
*Daphnia pulex*	10.00	500.00	9.20
*Daphnia (C.) similis*	56.25	100.00	7.28
*Ceriodaphnia dubia*	8.80	10.00	7.60

In our work, the higher sensibility at 48-h or more measured in *D. similis* can be explained by cumulative toxic effects: a mechanical toxic effect by adsorption/accumulation of large NPs aggregates due to its specific morphology and accompanied by a putative metabolic toxicity. In contrast, the lower sensibility showed by *D. pulex* can be explained by the metabolic toxicity alone as NPs only adsorb as small aggregates.

Consequently, we assume that the more important 48-h (or more) sensitivity of *D. similis* following CeO_2_ NPs exposure is due to the accumulation of aggregates that increase the drag force (decrease the swimming velocity). Large aggregates are however probably less efficient in inducing metabolic toxicity because these effects generally require a close proximity between the CeO_2_ NPs and the surface of the organism. This close proximity could explain the higher sensitivity at 24 h observed for *D. pulex* which only accumulates small aggregates close to the cuticle surface.

### 4.4. General Mechanistic Implications of CeO_2_ in Daphnia Physiological Functions

Among the different organism behavioral endpoints used to evaluate the risk associated to contaminants, the swimming performance of micro crustaceans is recognized particularly relevant, as this function is fundamentally correlated to numerous ecophysiological traits [33], [54]. The present work highlights that CeO_2_ NPs induce strong alteration of the daphnid swimming velocity related to the adsorption/accumulation of NPs onto the cuticle. Similar modifications of the swimming performance were observed in daphnids exposed to nC_60_, TiO_2_ and Ag NPs [32]–[35]. However, in these studies, no relationship between the NPs concentration and the alteration of the swimming behavior were measured/observed. Such concentration-response relationships were observed in studies dealing with the impact of dissolved metals and organic contaminants [20], [23], [25], [28]–[30]. To our knowledge, this work highlights for the first time the direct relationship existing between the decrease of the SV of daphnids and the existing concentration of NPs together with daphnid morphology effects.

Daphnids are filter feeders that are able to detect and migrate to food rich areas [55]. Thus a lower swimming capacity may directly impact their energy uptake and storage, and energetic metabolism. Our experiments showed that the hop frequency was not altered following exposure to NPs whereas the SV was dramatically decreased. This underlies that the daphnids attempt to maintain their swimming capacity but that the adsorption/accumulation of NPs onto their cuticles limit their movements through an increase of the viscous drag force. This might increase their energetic demand and lead to the organism death.

Another physiological parameter likely to be impacted by the decrease of the SV is the respiration rate. Daphnids generate a water current by swimming, this generates, through the carapace wall, gas exchange between the media and the haemolymph [56]. This water current also ensures a correct oxygenation of the eggs carried by mothers in their brood chambers [57]. An impaired capacity to swim decreases the water current, and consequently the O_2_ uptake by the organisms leading to anaerobiosis (*i.e.* a lower ATP supply).

All these sublethal effects related to swimming performance may impact survival capacities of the copepods exposed to CeO_2_ NPs.

### Conclusions

This work investigates the acute toxicity of CeO_2_ NPs in two species of daphnids focusing on the survival capacities and unusual (eco)toxicity endpoint, the swimming behavior. We observed strong interspecific differences in survival, adsorption of the NPs on the cuticle and the swimming performance. This highlights how important it is to compare different species in order to thoroughly understand and anticipate the ecotoxicological effects of NPs in the environment. However, in addition to the mechanistic effect underlined in the present work, further studies should explore the metabolic toxicity of CeO_2_ NPs in both species, such as oxidative stress, and ionic regulation that seems to be sensitive to the morphology and surface proximity of the CeO_2_ aggregates.
